# Thromboxane-Dependent Platelet Activation in Obese Subjects with Prediabetes or Early Type 2 Diabetes: Effects of Liraglutide- or Lifestyle Changes-Induced Weight Loss †

**DOI:** 10.3390/nu10121872

**Published:** 2018-12-02

**Authors:** Paola Simeone, Rossella Liani, Romina Tripaldi, Augusto Di Castelnuovo, Maria Teresa Guagnano, Armando Tartaro, Riccardo C. Bonadonna, Virginia Federico, Francesco Cipollone, Agostino Consoli, Francesca Santilli

**Affiliations:** 1 Department of Medicine and Aging and Center of Aging Science and Translational Medicine (CESI-Met), University of Chieti, 66100 Chieti, Italy; paolagsimeone@gmail.com (P.S.); rossellaliani@yahoo.it (R.L.); romina.tripaldi@hotmail.it (R.T.); guagnano@unich.it (M.T.G.); francesco.cipollone@unich.it (F.C.); consoli@unich.it (A.C.); 2 Department of Epidemiology and Prevention, IRCCS NEUROMED, Via dell’Elettronica, 86077 Pozzilli, Italy; dicastel@ngi.it; 3 Department of Neuroscience & Imaging, University of Chieti, 66100 Chieti, Italy; armando.tartaro@gmail.com; 4 Department of Medicine and Surgery, University of Parma, 43126 Parma, Italy; bonadonna.riccardo@fastwebnet.it; 5 Division of Endocrinology and Metabolic Diseases, Azienda Ospedaliera Universitaria of Parma, 43126 Parma, Italy; 6 Clinical Pathology, Chieti Hospital, 66100 Chieti, Italy; virgin83@virgilio.it

**Keywords:** obesity, diabetes mellitus, weight loss, oxidative stress, platelet activation, adipose tissue distribution, liraglutide

## Abstract

Thromboxane (TX)-dependent platelet activation and lipid peroxidation, as reflected in vivo by the urinary excretion of 11-dehydro-TXB_2_ and 8-iso-prostaglandin (PG)F_2α_, play a key role in atherothrombosis in obesity and type 2 diabetes mellitus (T2DM) since the earlier stages. Thirty-five metformin-treated obese subjects with prediabetes or newly-diagnosed T2DM were randomized to the glucagon-like peptide receptor agonist (GLP-RA) liraglutide (1.8 mg/day) or lifestyle counseling until achieving a comparable weight loss (−7% of initial body weight), to assess whether changes in subcutaneous (SAT) and visceral (VAT) adipose tissue distribution (MRI), insulin sensitivity (Matsuda Index) and beta-cell performance (multiple sampling OGTT beta-index), with either intervention, might affect TX-dependent platelet activation, lipid peroxidation and inflammation. At baseline, Ln-8-iso-PGF_2α_ (Beta = 0.31, *p* = 0.0088), glycosylated hemoglobin (HbA1c) (Beta = 2.64, *p* = 0.0011) Ln-TNF-α (Beta = 0.58, *p* = 0.0075) and SAT (Beta = 0.14, *p* = 0.044) were significant independent predictors of 11-dehydro-TXB_2_. After achievement of the weight loss target, a comparable reduction in U-11-dehydro-TXB_2_ (between-group *p* = 0.679) and 8-iso-PGF-_2α_ (*p* = 0.985) was observed in both arms in parallel with a comparable improvement in glycemic control, insulin sensitivity, SAT, high-sensitivity C-reactive protein (hs-CRP). In obese patients with initial impairment of glucose metabolism, the extent of platelet activation is related to systemic inflammation, isoprostane formation and degree of glycemic control and abdominal SAT. Successful weight loss, achieved with either lifestyle changes or an incretin-based therapy, is associated with a significant reduction in lipid peroxidation and platelet activation.

## 1. Introduction

Obesity is a risk factor for both diabetes (DM) and cardiovascular disease (CVD) [1]. Platelet activation and lipid peroxidation, as reflected in vivo by enhanced isoprostane generation and thromboxane (TX) biosynthesis, play a key role in the development of atherothrombosis in obesity and type 2 diabetes mellitus (T2DM) [2,3].

Our group was able to describe that in human metabolic disorders, such as obesity and DM, the underlying metabolic abnormalities may trigger inflammatory signals, with enhanced formation of reactive oxygen species (ROS), leading to increased lipid peroxidation and free radical–catalyzed conversion of arachidonic acid into bioactive isoprostanes. These may trigger and amplify platelet activation by activating the TX receptor in the presence of sub-threshold concentrations of other agonists [4].

We previously provided biochemical evidence of persistent TX-dependent platelet activation in obese women who are otherwise healthy and relatively young [5], and in newly diagnosed type 2 diabetic patients [6], whose TX biosynthesis was at least as high as previously reported in patients with longer-standing disease [2], supporting the hypothesis that platelet activation is related to the underlying metabolic disorder and not to vascular disease per se. This assumption is further substantiated by the linear correlation between the urinary excretion of the major TX metabolite, 11-dehydro-TXB_2_ and either body mass index (BMI) or glycemic control, as reflected by fasting and postprandial plasma glucose or HbA1c. Consistently, several lifestyle and pharmacological interventions targeting the culprit metabolic perturbation, namely decrease in body weight and improvement in glycemic control, respectively, have been previously associated with the reversal of these biochemical abnormalities associated with both obesity and DM, thus strengthening the dependence of platelet activation on adiposity and hyperglycemia, in the case of obesity and DM, respectively [5,6]. Until now, the relative contribution of adiposity, adipose tissue inflammation, insulin resistance, and beta cell deterioration with consequent hyperglycemia to persistent platelet activation, has been difficult to dissect. Whether TX-dependent platelet activation is sustained by different metabolic triggers in different phases along the line linking obesity to overt T2DM, is still an open and relevant question, since hyperglycemia is a weak risk factor for CVD [7,8], and interventions [9,10,11] focused on reducing plasma glucose have failed to significantly reduce CV risk and mortality.

In this regard, an important source of missing information may potentially come from the setting of prediabetes, where the underlying metabolic abnormalities, a variable combination of impaired insulin secretion, insulin resistance, low-grade inflammation, abnormal body fat distribution, do not include overt hyperglycemia [12].

Liraglutide, an analog of the incretin hormone, glucagon-like peptide 1 (GLP-1), initially used for the treatment of T2DM, has recently been introduced as potential weight loss medication, since it has been shown to delay gastric emptying and induce satiety, leading to decreased energy intake and weight reduction [13]. It stimulates a decrease in blood glucose levels by increasing the amount of insulin released from pancreatic beta cells after eating, prior to the elevation of blood glucose levels [14]. Liraglutide has been recently reported to inhibit platelet activation in animal models [15] and in healthy volunteers, by increasing nitric oxide (NO) effects [16]. 

In a group of obese subjects with prediabetes or early T2DM randomized to liraglutide or lifestyle changes to achieve comparable weight loss, we recently observed significantly enhanced abdominal visceral fat loss and improved beta-cell function with liraglutide [17]. Both interventions were equally effective on subcutaneous fat loss, systemic inflammation, and on glycemic control, albeit with a greater effect of liraglutide on glucose tolerance. In the same population, we intended to investigate and dissect out the relative contribution of inflammation, adipose tissue distribution, insulin resistance, and beta cell deterioration to persistent platelet activation, and evaluate, at equal degree of weight loss, if a treatment by a GLP-1 receptor agonist (GLP-1 RA) exert a greater impact than lifestyle changes on TX-dependent platelet activation and lipid peroxidation. 

## 2. Materials and Methods 

### 2.1. Subjects and Study Design

This study was part of a longitudinal, randomized, controlled, parallel-arm study designed to assess, in obese subjects with impaired glucose tolerance (IGT) and/or impaired fasting glucose (IFG) or early T2DM, the effects of an equal degree of weight loss, achieved by either lifestyle changes or liraglutide, on cardiometabolic variables [17]. Each subject signed written informed consent to participate, and the Protocol was approved by the Ethics Committee of the University of Chieti (Approval n. 10 (protocol 20131) 23.05.2013). Enrollment took place at the Obesity and Diabetes Clinics of Chieti University Hospital. All study visits were performed at the Clinical Research Center (CRC) within the Center of Aging Science and Translational Medicine (CESI-Met), “G. d’Annunzio” University Foundation.

As previously published [17], we enrolled obese subjects (BMI ≥ 30), with a diagnosis of IGT or IFG or T2DM for less than 12 months, according to the American Diabetes Association (ADA) Guidelines [18], under diet therapy associated with ongoing metformin treatment at the highest tolerated dose (up to 3000 mg/day) at the time of enrolment. 

Exclusion criteria included type 1 DM, diagnosed with islet autoantibodies evaluation (islet cell cytoplasmic, and islet antigen 2 (IA-2) antibodies, anti-glutamic acid decarboxylase), when one of the following applied: age lower than 40, family history of type 1 DM, lean phenotype, early requirement for insulin; MODY (Maturity Onset Diabetes of the Young). Additional exclusion criteria were BMI < 30, DM diagnosis longer than 12 months, oral antidiabetic agents (except metformin) or insulin treatment in the last three months, uncontrolled hypertension (systolic/diastolic blood pressure > 160/90 mmHg), significant comorbidities including kidney disease (glomerular filtration rate below 60 mL) or liver disease (aspartate aminotransferase (AST) or alanine aminotransferase (ALT) twice above the upper normal range), pregnancy or lactation; sexually active female of child-bearing potential not using adequate contraceptive methods; chronic non-steroidal anti-inflammatory drug therapy; any contraindication to liraglutide (known or suspected hypersensitivity to GLP-1 receptor agonists, previous acute or chronic pancreatitis, inflammatory bowel disease, gastrointestinal surgery, heart failure Class NYHA III-IV); personal or family history of medullary thyroid carcinoma or of multiple endocrine neoplasia type 2 (MEN2); claustrophobia; metal implants or other contraindications for magnetic resonance imaging (MRI); recent participation in other research projects within the last 3 months or participation in 2 or more projects in one year.

After a baseline evaluation, the patients were randomized in a 1:1 ratio to receive liraglutide or lifestyle counselling. Study medication (liraglutide 6.0 mg/mL in 3-mL prefilled pen injectors) was supplied by Novo Nordisk. Liraglutide was administered by daily subcutaneous injection at bedtime and titrated over a 3-week period: 0.6 mg per day during the first week, 1.2 mg daily (second week), and 1.8 mg daily (third week), based on the clinical response and side effects. The non-attainment of the 1.8 mg dose was not a withdrawal criterion. Patients in the Liraglutide arm received some advises on physical activity and diet, without a structured intervention program.

The computer-generated random allocation sequence was prepared by the trial statistician in blocks of four participants. The subjects were assigned a consecutive random number based on the order of inclusion in the study, and were then allocated to one of the two treatment groups.

The weight loss goal for all the participating subjects was to lose 7% of initial body weight (calculated at the time of randomization). This weight loss goal was established based on the observation that this amount of weight reduction was associated with improved metabolic outcomes [19] and with reduced TX-dependent platelet activation in obese women [5,20].

Patients not achieving the weight loss goal within 15 months of the initiation of the randomized treatment, as well as those not completing the study for decision of the patient and/or of the investigator, were considered withdrawn from the study, and were replaced, in order to attain the anticipated sample size.

All subjects, after signing the informed consent, underwent, at baseline and after the end of the randomized treatment: clinical evaluation; abdominal MRI for the assessment of adipose tissue (AT) distribution in terms of visceral AT (VAT) and subcutaneous AT (SAT) [21]; oral glucose tolerance test (OGTT) with frequent sampling, after 12-h overnight fast, for assessment of insulin sensitivity and beta cell function [22]; measurement of the urinary excretion of 8-iso-PGF_2α_ and 11-dehydro-TXB_2_, reflecting in vivo lipid peroxidation and platelet activation, respectively; blood sampling in the fasting state for measurement of fasting plasma glucose, fasting insulin, circulating levels high-sensitivity C-reactive protein (hsCRP), insulin-like growth factor-I (IGF-I), leptin, tumor necrosis factor-α (TNF-α).

Periodic visits, every 3 weeks, were planned to reinforce the motivation to achieve the weight loss goal, by monitoring compliance to liraglutide (by pill counting) or to lifestyle changes (see below). At each visit, participants completed questionnaires and underwent physical examination. Each patient was carefully monitored for adverse events. 

#### 2.1.1. Lifestyle Intervention Program

Participants in the lifestyle intervention arm were encouraged to achieve the 7% weight loss in the first 6 months, based on previous studies suggesting that most subjects achieve their maximum weight loss within the first 20–24 weeks of a lifestyle program. Recommendations were provided as written information and periodic 20-to-30-min individual sessions were prescribed in order to emphasize the importance of a healthy lifestyle. Visits with the staff of nutritionists were planned once a week during the first 4 weeks, then once every 2 weeks for the following 20 weeks, finally once a month. Participants were encouraged to follow the Food Guide Pyramid and a healthy low-calorie, low-fat diet, the equivalent of a National Cholesterol Education Program Step 1 diet, to lose weight, and to increase the intensity and frequency of their physical activity to moderate intensity (such as brisk walking) for at least 150 min per week, to achieve at least 700 kcal/week expenditure. The mean caloric intake before randomization was 2050 kcal, with an average fiber consumption of less than 24 g/day, proteins 17%, lipids 32% (10% of which saturated fat, 9% polyunsaturated fat, 12% monounsaturated fat), carbohydrates 51% (14% of which is simple sugars) of energy intake.

The caloric content of the food plan has been adapted and customized taking into account the following aspects: Age, sex, body weight, stature, working activity, physical activity level, considering a range between a sedentary hypokinetic profile and a profile with marked motor involvement. Given these factors, the Average Energy Requirement was calculated considering the Basal Metabolism (Harris-Benedict Formula) of the subjects and the physical activity level. The expected caloric intake was 1200–1800 kcal per day (Average 1450 kcal).

The calories were divided on average as follows: 15% proteins; 30% fat of which 8% was saturated fat, 12% monounsaturated fatty acids (MUFA), 8% polyunsatured fatty acids (PUFA), 55% carbohydrate, 10% of which was simple sugars. The amount of fiber had to be 25 g/day. Daily water intake had to be not less than 1.5 L.

Before the randomization, only 3 patients performed structured exercise (pool or exercise bike for 1 h, 3 times a week). The remaining patients practiced unstructured physical activity such as walks of varying duration and frequency. 

#### 2.1.2. Oral glucose tolerance test (OGTT) with Frequent Sampling

Subjects underwent OGTT with frequent sampling before and after the foreseen weight loss, after 36 h since the last administration of liraglutide for those in the liraglutide arm, as previously described [17]. The patients were instructed to consume a weight-maintaining diet containing 200–250 g of carbohydrate per day for at least 3 days before the OGTT. They were admitted to the CRC at 8 am after 10–12 h overnight fast. For post-weight loss OGTT, liraglutide was withheld on the evening before the OGTT such that the last dose was administered approximately 36 h earlier. Each study lasted 130 min (–10 s to 120 s). At time 0 s, subjects ingested a 75-g glucose solution over 5 s. Blood samples was collected at −10 s, 0 s, 15 s, 30 s, 45 s, 60 s, 90 s, 120 s to measure plasma glucose and serum C-peptide and insulin (baseline samples and +30 s) concentrations. 

Insulin sensitivity was obtained using the Matsuda index, which reflects a composite of both hepatic and peripheral tissue insulin sensitivity, as previously described [23].

Beta cell function during OGTT was estimated by applying to the glucose and C-peptide curves of each subject a minimal model of glucose-induced insulin secretion and computing the OGTT beta-index, as previously described in detail [22].

#### 2.1.3. Magnetic Resonance Imaging (MRI) Quantification of Visceral and Subcutaneous Fat

A Achieva Philips 1.5 Tesla body scanner (Amsterdam, The Netherlands), available at the Institute for Advanced Biomedical Technologies (ITAB), a neuroscience and imaging research center within the University of Chieti “G. d’Annunzio”, was employed to obtain magnetic resonance (MR) images. All acquisitions were obtained through a spin-echo sequence with a 500-ms repetition time and 20-ms echo time. To plan the data acquisition, a transverse and sagittal image of the abdomen region were taken to identify the intervertebral space between the lumbar fourth (L4) and fifth (L5) vertebrae. Transverse slices (10 mm thick) were then acquired every 50 mm, from the L4–L5 space toward the feet. The optimal threshold for adipose tissue was 110 (on a scale of 256). Adipose tissue area and volume were calculated as previously described [21]. 

### 2.2. Analytical Measurements

#### 2.2.1. Biological Material Collection

At admission to the study and after the achievement of the weight loss goal, venous blood samples were collected and frozen at −20 °C for subsequent biochemical measurements. All subjects were studied as out-patients after a 12-h fast and overnight urine collection was performed immediately before blood sampling. Urine samples were added with the antioxidant 4-hydroxy-Tempo (1 mM) (Sigma Chemical Co., St. Louis, MO, USA) and stored at –20 °C until extraction.

#### 2.2.2. Biochemical Measurements

Plasma glucose concentration was determined by the glucose oxidase method and serum insulin and C-peptide levels by immunochemiluminometric assays. The HbA1c was measured by automated high-performance liquid chromatography (HPLC) [24]. The homeostasis model assessment of insulin resistance (HOMA-IR) was calculated as described by Matthews et al. [25]. Serum hs-CRP concentrations were measured using highly sensitive immunoassay. Serum insulin-like growth factor I (IGF-I) was measured with specific radioimmunoassay kits (Mediagnost, Tubingen, Germany), as previously described [26]. Plasma leptin and TNF-α were measured by enzyme-linked immunosorbent assays (ELISA) purchased from R&D (DLP00 and HSTA00D respectively, Minneapolis, MN, US).

#### 2.2.3. Urinary Eicosanoid Assays

Urinary 8-iso-PGF_2α_ (U-8-iso-PGF_2α_) and 11-dehydro-TxB_2_ were measured by previously described radioimmunoassay methods that have been validated by using different antisera and by comparison with gas chromatography/mass spectrometry [27,28].

### 2.3. Statistical Analysis

This is a post hoc analysis of a published study [17]. The sample size calculation for the present sub-study has been performed as a post-hoc power assessment. The available sample size ensures a 90% power (alpha = 0.05) to detect between liraglutide and lifestyle intervention, at the end of the treatment period, a mean difference in 11-dehydro-TxB_2_ values of at least 1 standard deviation (of the distribution of 11-dehydro-TXB_2_ changes).

In the present analysis of the study, the outcome of interest was the change in urinary 11-dehydro-TxB_2_ (U-11-dehydro-TXB_2_) and 8-iso-PGF_2α_ after achievement of 7% of initial weight loss. Secondary outcomes were changes in metabolic, biochemical and imaging parameters in study.

The Kolmogorov–Smirnov test and examination of residual distribution were used to determine whether each variable has a normal distribution. When necessary, natural-log transformation was used to normalize the data, or appropriate non-parametric tests were used. Comparisons of baseline data between the groups were performed by chi-squared statistics, Fisher exact tests, unpaired Students *t*-tests or Mann–Whitney *U*-tests.

For comparison between arms of the study, we used a linear mixed-effects model for repeated measures over time, with delta U-11-dehydro-TXB_2_ as the dependent variable, study group and time-by-group interaction as fixed effects, time-to-weight loss (month), basal weight, waist and VAT levels as fixed effect covariates and patients and error as random effects. Within the mixed model, we obtained least-squares estimates of the treatment differences and standard errors, and estimated 95% confidence intervals (CIs) and *p*-values for the two pre-specified intergroup contrasts (liraglutide and lifestyle intervention) for baseline and end of study within each group. For other continuous variables, we used the same procedure.

Univariable correlation test and multivariable linear regression analysis were used to assess relationship among continuous variable at baseline and to characterize predictors of change in U-11-dehydro TXB_2_ after weight loss.

*P*-values lower than 0.05 were regarded as statistically significant. A two-sided *p*-value <0.05 was considered as statistically significant. The data analysis was generated using SAS/STAT software, Version 9.1.3 of the SAS System for Windows^©^2009 (SAS Institute Inc. Cary, NC, USA).

This study was performed under the Good Clinical Practice regulations (Good Clinical Practice for Trial on Medicinal Product-CPMP/European Commission -July 1990; Decreto Ministeriale 27.4.1992-Ministero della Sanità) and the Declaration of Helsinki (Hong Kong 1989). By signing the protocol, the participants in the study committed to adhere to local legal requirements.

## 3. Results

One-hundred and twenty-two patients consecutively referred to the outpatient Diabetes Clinic and Obesity Centre of our University Hospital were assessed for eligibility between June 2012 and September 2013. Among those, 70 were excluded (50 did not meet the eligibility criteria, 10 refused to participate, 8 had claustrophobia, and 2 had a pacemaker). Sixty-two patients were randomized to one of the two treatment arms—31 were allocated to liraglutide and 31 to lifestyle changes—and monitored until achievement of the weight loss goal. Participants were enrolled from October 2013 to July 2015.

Twenty-two subjects were excluded from the study. Of them, 10 did not achieve the specified weight loss goal within the allowed 15-month period, 12 were lost to follow-up (9 for unwillingness to continue the study, 1 for metformin intolerance, 1 for pregnancy, 1 for severe anemia). Five additional patients were excluded from the analysis of the present study because of ongoing antiplatelet treatment (3 since baseline, 2 initiated low-dose aspirin during the study), which is known to influence the urinary levels of 11-dehydro-TXB_2_ [2,3,4]. Completers were comparable to non-completers with regard to baseline characteristics (data not shown) [17]. 

**Figure 1:**
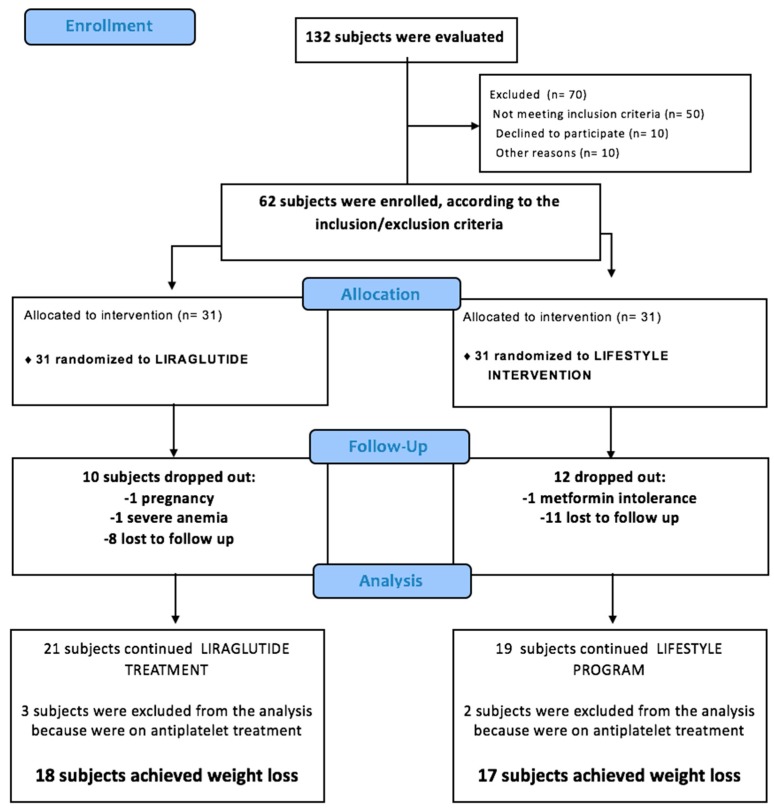
Flow diagram of the patients included in the study.

No serious adverse event took place during the treatment period (Figure 1).

Thus, urinary eicosanoid excretion was evaluated on 35 patients (Table 1). Median time-to-weight loss was 4 months (4 (3–6) months), without any difference between the two arms (4.0 (3.2–6.0) months and 4.0 (3.0–6.0) months in the liraglutide and lifestyle arms, respectively).

Table 1 shows the clinical and biochemical characteristics of the 35 patients who completed this study, stratified according to the treatment arm. Of the whole population, 20 subjects had prediabetes, 15 newly diagnosed T2DM.

### 3.1. Baseline Evaluation

**Figure 2:**
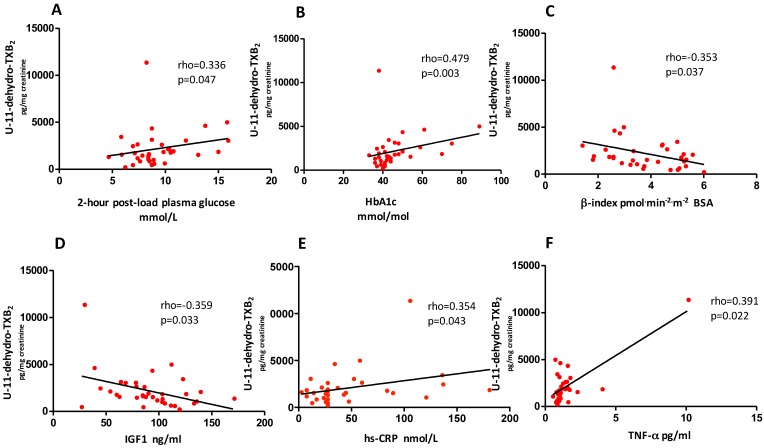
Baseline correlations between platelet activation and metabolic control, beta cell function, IGF-I, and systemic inflammation in obese patients with prediabetes or early type 2 diabetes. Basal correlations between U-11-dehydro-TXB_2_ and 2-h post-load plasma glucose (panel **A**), HbA1c (panel **B**), beta cell function as assessed by beta index (panel **C**), IGF-I (panel **D**), systemic inflammation as assessed by hs-CRP (panel **E**) and TNF-α (panel **F**) in obese patients with prediabetes or newly diagnosed type 2 diabetes. IGF-I, insulin growth factor-I, hs-CRP, high sensitivity C-reactive protein, TNF, tumor necrosis factor.

**Figure 3:**
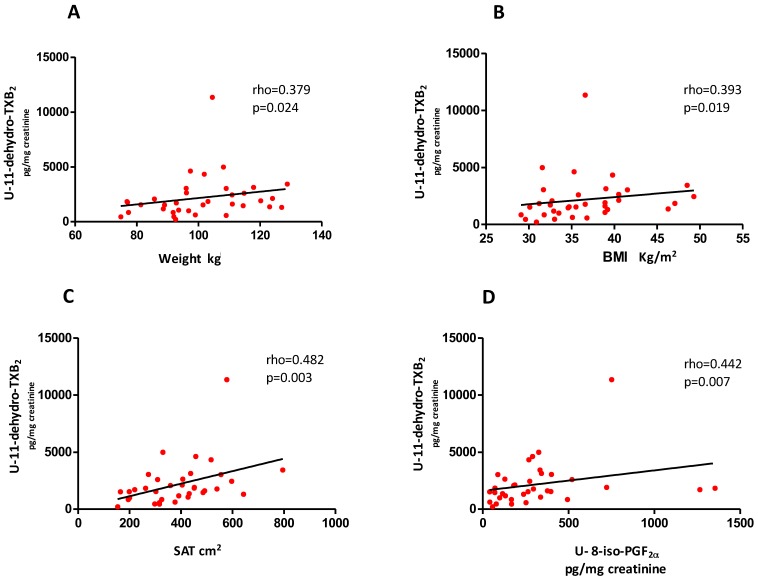
Baseline correlations between platelet activation and weight, body mass index (BMI), subcutaneous adipose tissue area, and lipid peroxidation. Basal correlation between U-11-dehydro-TXB_2_ and weight (panel **A**), BMI (panel **B**), SAT (panel **C**), and U-8-iso-PGF_2α_ (panel **D**) in obese patients with prediabetes or early diabetes. SAT, subcutaneous adipose tissue.

At baseline, no difference was observed either in U-11-dehydro-TXB_2_ (*p* = 0.0989) or in U-8-iso-PGF_2α_ (*p* = 0.9336) between subjects with prediabetes and diabetes. U-11-dehydro-TXB_2_ was directly related to 2-h post-load plasma glucose (rho = 0.336, *p* = 0.047) and HbA1c (rho = 0.479, *p* = 0.003), and inversely related to beta cell function, as assessed by OGTT beta-index (rho= −0.353, *p* = 0.037) and to IGF-I (rho = −0.359, *p* = 0.033) (Figure 2, panels A–D). U-11-dehydro-TXB_2_ was also directly related to hs-CRP (rho = 0.354, *p* = 0.043), and TNF-α (rho = 0.391, *p* = 0.022), reflecting systemic inflammation (Figure 2, panels E–F). Moreover, platelet activation was correlated directly with weight (rho = 0.379, *p* = 0.024), BMI (rho = 0.393, *p* = 0.019), SAT (*r* = 0.482, *p* = 0.003), but not VAT, and U-8-iso-PGF_2α_ (rho = 0.442, *p* = 0.007), reflecting in vivo lipid peroxidation (Figure 3, panels A–D and data not shown). Interestingly, SAT but not VAT was directly related to hs-CRP (*r* = 0.417, *p* = 0.015) (data not shown). Noteworthy, one subject, who was an outlier for U-11-dehydro-TXB_2_, with particularly high urinary thromboxane metabolite excretion, also showed very high levels of markers of inflammation (hs-CRP, TNF-α) and very low circulating IGF-I.

On multivariable regression analysis, baseline Ln-8-iso-PGF_2α_ (Beta = 0.31, SE = 0.10, *p* = 0.0088), HbA1c (Beta = 2.64, SE = 0.69, *p* = 0.0011) Ln-TNF-α (Beta = 0.58, SE = 0.19, *p* = 0.0075) and SAT (Beta = 0.14, SE = 0.06, *p* = 0.044) were significant independent predictors of thromboxane-dependent platelet activation (Table 2).

### 3.2. Effects of Liraglutide and Lifestyle Interventions

**Figure 4:**
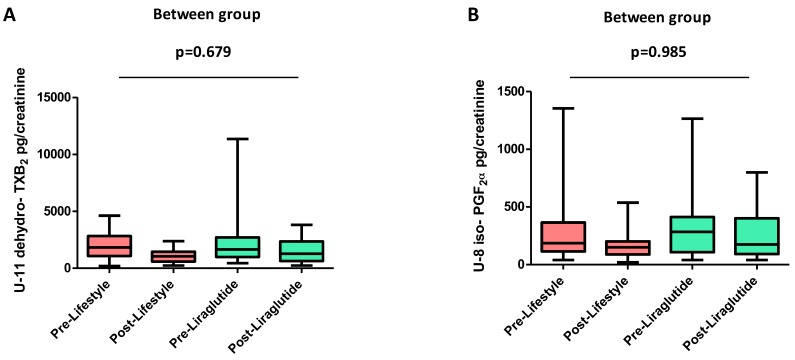
Effects of Liraglutide- or lifestyle-induced weight loss on platelet activation and lipid peroxydation in obese subjects with prediabetes or early type 2 diabetes mellitus. Effect of liraglutide- or lifestyle counseling–induced weight loss on in vivo platelet activation, as assessed by U-11-dehydro-TXB_2_ (panel **A**), and in vivo lipid peroxidation, as assessed by U-8-iso PGF_2α_ (panel **B**), in obese patients with prediabetes or diabetes diagnosed within one year.

At baseline, the only significant between-arm differences were higher waist (*p* = 0.021) and lower VAT (*p* = 0.027) in the liraglutide arm than in the lifestyle arm (Table 1), thereby the main analysis was adjusted for these baseline variables. The between-arm difference in waist disappeared when adjusting for VAT (*p* = 0.20). After achievement of the weight loss target (−7% of the initial body weight) in the two groups, a significant reduction in U-11-dehydro-TXB_2_ was observed in both arms, with no difference between arms (median percent change vs. baseline −24.1% vs. −24.3% in the liraglutide and lifestyle arms, between-group *p* = 0.679, Figure 4A), in parallel with a comparable improvement in glycemic control, insulin sensitivity, SAT, hs-CRP (between-arm *p* = ns), as previously reported [17]. A non-significant reduction in urinary 8-iso-PGF-_2α_ occurred with both interventions, without differences between arms (−14.6% vs. −21.2%, between-group *p* = 0.985, Figure 4B). Finally, after comparable weight loss, serum TNF-α was not significantly reduced in either arm.

### 3.3. Predictors of Change in U-11-Dehydro-TXB_2_ after Weight Loss

The lack of treatment-effect on U-11-dehydro-TXB_2_ and 8-iso-PGF-_2α_ allowed us to evaluate the effect of weight loss per se, regardless of the intervention arm, in the whole cohort of 40 studied patients. U-11-dehydro-TXB_2_ and U-8-iso PGF_2α_ were both significantly reduced after achievement of the weight loss target (Table 3).

Univariate correlations between change in U-11-dehydro-TXB_2_ and changes in other variables after weight loss are reported in Table 4. On multivariable regression analysis, reduction in U-8-iso-PGF_2α_ (Beta = 0.23, SE = 0.23, *p* = 0.0042), and in TNF-α (Beta = 0.10, SE = 0.04, *p* = 0.029) were the only independent predictors of U-11-dehydro-TXB_2_ decrease in the whole group after weight loss (Table 5).

## 4. Discussion

Atherothrombosis is a common and somehow unpredictable complication of diseases associated with abnormalities in glucose metabolism, namely prediabetes and DM, its occurrence being not strictly related to the degree of metabolic impairment or to disease duration, as observed instead with regard to microvascular complications [29]. Consistently, lowering HbA1c has only a modest effect on reducing CVD risk and mortality [7,11], whereas newer drugs, such as liraglutide, semaglutide and empagliflozin have proved effective in reducing CVD risk beyond glucose lowering [30,31,32], thus suggesting that different drug targets need to be modulated in order to blunt atherothrombosis [33].

Persistent platelet activation has been regarded as a pivotal link between metabolic abnormalities and accelerated atherogenesis and thrombosis in these settings. The observation that biochemical evidence of TX-dependent platelet activation may be detectable as early as within one year since the diagnosis of T2DM [6], together with its correlation with glycemic control and reversal with its improvement [2,3], point-out to hyperglycemia, both fasting and postprandial, as the main trigger for the activation of platelets in this setting. However, the pathophysiological soil underlying the pathogenesis of T2DM, as exemplified by the ominous octet [34], is far more complex, and the relative contribution of each pathogenic component to persistent platelet activation is difficult to dissect. In this regard, prediabetes may represent a suitable model in that it is not characterized by overt hyperglycemia, which is the final consequence of insulin resistance as well as impaired insulin secretion. Prediabetes has been associated with a 40% reduction in whole-body insulin sensitivity, substantial decline in glucose sensitivity of β cells, and increased waist circumference and BMI when compared with normal glucose tolerance [35]. However, prediabetes is a highly heterogeneous metabolic state, both with respect to its pathogenesis and prediction of disease [36], where a variable combination of the above-mentioned factors may contribute to the likelihood to develop vascular complications [37]. 

To address the issue of the metabolic determinants of platelet activation, we designed an investigative approach with the following features: (i) inclusion of patients with prediabetes, to evaluate the occurrence of TX-dependent platelet activation and its potential determinants in the absence of overt hyperglycemia; (ii) accurate clinical characterization of patients, by employing state-of-the art assessment tools including abdominal MRI for adipose tissue distribution, OGTT with frequent sampling allowing mathematical models to predict insulin sensitivity and beta-cell function; (iii) a randomized design of the study, controlled with a different intervention leading to the same degree of weight loss, to dissect out the relative contribution of liraglutide per se, regardless of the concurrent weight loss effect, on study endpoints.

In our study, we showed biochemical evidence of persistent TX-dependent platelet activation in obese patients with prediabetes or newly diagnosed T2DM, in good metabolic control. More importantly, we established that average glucose levels, as reflected by HbA1c, and impaired beta cell function, in the absence of overt hyperglycemia, are potential metabolic triggers for platelet activation in this setting. Of note, TX metabolite excretion was inversely correlated with both post-challenge insulin and c-peptide values on multiple sampling (data not shown). A decreased incretin (GLP-1/glucose-dependent insulinotropic polypeptide (GIP)) effect resulting from impaired GLP-1 secretion [38] may be advocated as one of the possible underlying mechanisms fostering the activation of platelets. Consistently, GLP-1R activation in platelets has been shown to attenuate platelet aggregation and thrombosis both in mice and in humans [15,16]. *Vice versa*, activated platelets may promote cell dysfunction through the release into the circulation of inflammatory citokines, such as TNF superfamily member 14 (LIGHT/TNFSF14), which impairs insulin secretion in pancreatic islet cells, as shown by our group and others [39]. Thus, a bidirectional link between beta cell dysfunction and platelet activation may exist, largely mediated by inflammation.

Indeed, besides metabolic control, systemic inflammation, as reflected by circulating TNF-α, as well as oxidative stress, as reflected by isoprostane biosynthesis, were strong independent predictors of TX biosynthesis, thus confirming the large body of in vivo evidence, provided by our group and others, of metabolic abnormalities triggering inflammatory signals, with enhanced reactive oxygen species (ROS) formation, leading to increased lipid peroxidation and free radical–catalyzed conversion of arachidonic acid into bioactive isoprostanes, able to activate TX receptor [2,3,4,5,40]. Of interest is the counterintuitive finding that the extent of SAT, unlike VAT, is related and is possibly a determinant of platelet activation. This finding is, in appearance, in contrast to our previous results of a higher degree of TX-dependent platelet activation in android obesity, defined as waist hip ratio (WHR) higher than 0.86, roughly reflecting visceral or central obesity [5]. However, the accuracy of MRI in the detection of abdominal subcutaneous fat as opposed to visceral fat is much higher than the antropometric estimate [41], and no study until now had evaluated the relationship between adipose tissue distribution and platelet activation. Surprisingly, in our study, SAT but not VAT was linearly and directly related to hs-CRP, suggesting that SAT may be an additional important source of systemic inflammation which in turn may trigger lipid peroxidation and platelet activation. Indeed, the expression of critical pro-inflammatory genes is substantially higher in SAT than in VAT in individuals with morbid obesity [42]. Moreover, among healthy subjects, SAT was the most consistent indicator for increased levels of hs-CRP [43]. Both baseline VAT volume and SAT volume as well as their increase over time were associated with incident metabolic risk factors beyond overall adiposity among participants from the Framingham Heart Study [44,45,46]. In addition, a net release of IL-6 by subcutaneous abdominal tissue has been shown to occur in vivo in humans [47], and this release may be postulated to be one of the determinants of CRP release by the liver. Finally, primary subcutaneous adipose tissue has been shown to generate megakaryocytes and platelets, suggesting a link between the activation of SAT and platelet production and activation, although the underlying molecular mechanisms are still unravelled [48]. 

Thus, even in the absence of overt hyperglycemia and poor glycemic control, inflammatory triggers, likely derived from abdominal subcutaneous adiposity, may promote beta cell deterioration, an early event preceding the clinical diagnosis of DM, ultimately leading to platelet activation. The understanding of these intertwined mechanisms provides the rationale for implementing strategies to revert platelet activation by targeting one or more of the various components along the depicted pathophysiological line, from beta cell function and adiposity to inflammation, oxidative stress and platelet activation. In addition to antiplatelet agents, acting downward this cascade, novel drugs for the treatment of DM, namely GLP-1 RA, are of interest since they have been reported to revert beta cell function both in animal models, by inhibiting pancreatic beta-cell apoptosis and stimulating the proliferation and differentiation of beta-cells [49], and in human studies [17,50].

Along these lines, beyond improvement in glycemic control and weight loss, platelet inhibitory effects have been hypothesized for GLP-1RAs in both experimental models [15,51] and in healthy volunteers [16].

Thus, liraglutide has the potential, at least theoretically, to modulate platelet activation both directly, through its effects on platelet function, and indirectly, through its effects on body weight and metabolic control, and specifically on beta cell function.

Until now, several lifestyle and pharmacological interventions, namely insulin, acarbose, pioglitazone, rosiglitazone, have been previously associated with reversal of inflammation, lipid peroxidation and TX-dependent platelet activation in the settings of obesity or T2DM, in parallel with a variable decrease in body weight and/or improvement in metabolic control [2,3,5,6,17,52,53,54].

Thus, we performed a randomized study, controlled with a different intervention leading to the same degree of weight loss, to dissect the relative contribution of liraglutide per se, regardless of the concurrent weight loss effect, on U-11-dehydro-TXB_2_ excretion rate.

Against our hypothesis, lifestyle intervention and liraglutide therapy were equally effective to reduce the levels of U-11-dehydro TXB_2_, as well as U-8-iso-PGF_2α_. Despite the observed greater improvement in beta-cell function with liraglutide [17] and the putative role of this drug on platelets, as suggested by other authors [16], liraglutide-induced weight loss did not exert a significantly greater effect on TX biosynthesis as compared to lifestyle changes-associated weight loss.

Thus, regardless of the type of treatment, weight loss per se, together with concurrent improvement of glycemic control, was associated with changes in 11-dehydro-TXB_2_ and 8-iso-PGF_2α_ in obese subjects with prediabetes or early type 2 diabetes. This finding substantiates current guidelines in this setting, advocating lifestyle changes as the first line strategy for the prevention of diabetes and its complications [55].

Interestingly, in the whole group of evaluated patients, reduction in urinary 8-iso-PGF_2α_ and in TNF-α were independent predictors of the decrease in TX-dependent platelet activation. Thus, the effect of weight loss on TX metabolite excretion, whatever the intervention, may be mediated by a favorable impact on inflammation and lipid peroxidation. 

Several limitations should be acknowledged. First of all, the relatively limited sample size, which might have prevented detection of differences between the two arms; second, the length of exposure to interventions was relatively low (median, interquartile range (IQR), 4 (3–5.75) months). We cannot exclude that longer treatment durations might have resulted in different findings. Third, a prerequisite to detect differences in our study is that weight loss and GLP-receptor engagement by liraglutide are additive to each other as to their effects on the endpoints. Thus, our results do not exclude the hypothesis that, in patients experiencing a 2–3 kg or no weight loss, liraglutide can affect inflammatory and/or platelet biomarkers. In addition, subjects not achieving the weight loss goal were not re-evaluated, thus preventing any information on the effects of liraglutide in the absence of weight loss. Of note, the time frame between last liraglutide administration and urine collection suggests that our measurements of platelet activation and lipid peroxidation were performed at a time when the plasma concentrations of liraglutide were lower than those achieved during the steady state [56]. In this regard, further studies will be needed to assess whether liraglutide exerts any effect on platelets, its dose-dependence, and whether this effect is transient or cumulative on repeated daily dosing.

On the lifestyle intervention side, patients randomized to the lifestyle arm underwent a structured lifestyle program designed to improve adherence and to better tailor interventions on the single individual. A similar program is not easily transposable into every day practice, mainly because it is time-consuming for the practicing physician and requires dedicated personnel.

## 5. Conclusions

In conclusion, in obese patients with initial impairment of glucose metabolism, the extent of platelet activation is related to the degree of inflammation and lipid peroxidation, as well as to metabolic control and adipose tissue distribution. Successful weight loss, achieved with either lifestyle changes or an incretin-based therapy, is associated with a significant reduction in TX-dependent platelet activation, possibly mediated, at least in part, by decreased inflammation and lipid peroxidation. Further studies are needed to address the question whether a longer-term, or higher dose liraglutide treatment, or a combined strategy coupling diet, exercise and liraglutide, as suggested by the guidelines [55], may exert more powerful effects on TX-dependent platelet activation. Meanwhile, lifestyle interventions could be regarded as first-line strategies for the prevention of cardiometabolic diseases in obese patients with either prediabetes or early, overt T2DM.

## Figures and Tables

**Table 1:** Clinical and laboratory baseline characteristics of obese patients randomized to Liraglutide- or lifestyle-induced weight-loss intervention.

Variable	Pre-Liraglutide (*n* = 18)	Pre-Lifestyle (*n* = 17)	*p*-value *
**Age (years)**	55.5 (48.0–64.0)	53.0 (51.0–55.0)	0.753
**Gender (male), *n* (%)**	11 (61)	8 (47)	0.505
**BMI (kg/m^2^)**	36.6 (34.6–39.0)	33.5 (31.2–39.8)	0.222
**Type 2 diabetes, *n* (%)**	8 (44)	7 (41)	-
**IGT/IFG, *n* (%)**	10 (59)	10 (56)	-
**Waist (cm)**	114.5 (112.0–127.0)	105.0 (100.0–117.0)	0.021
**Systolic BP (mmHg)**	146.0 (130.0–154.0)	137 (123–144)	0.120
**Diastolic BP (mmHg)**	84.0 (79.0–88.0)	80 (72–84)	0.124
**Smoke, *n* (%)**	4 (22)	0 (0)	0.153
**Hypertension, *n* (%)**	15 (83)	9 (53)	0.075
**Dyslipidemia, *n* (%)**	7 (39)	8 (47)	0.738
**CVD, *n* (%)**	1 (5.6)	3 (17.6)	0.337
**Previous MI, or revascularization, *n* (%)**	0 (0)	0 (0)	-
**Previous TIA/stroke, o revascularization, *n* (%)**	0 (0)	1 (5.9)	0.485
**PAD, *n* (%)**	1 (5.6)	0 (0)	-
**Carotid stenosis, *n* (%)**	1 (5.6)	4 (23)	0.177
**Microvascular disease, *n* (%)**	0 (0)	1 (5.9)	0.485
**Total cholesterol (mmol/L)**	4.3 (3.6–4.8)	4.4 (4.0–4.6)	0.632
**High density lipoprotein (HDL) cholesterol (mmol/L)**	1.1 (1.0–1.4)	1.1 (1.0–1.3)	0.934
**Triglycerides (mmol/L)**	1.4 (0.9–2.3)	1.1 (0.8–1.3)	0.116
**Amylase (U/L)**	60.5 (55.0–71.0)	64.0 (52.0–75.0)	0.973
**Lipase (U/L)**	106.0 (76.0–118.0)	118.0 (71.0–156.0)	0.241
**Fasting plasma glucose (mmol/L)**	5.1 (4.9–5.9)	5.3 (5.1–5.7)	0.791
**1-h post load plasma glucose (mmol/L)**	10.6 (9.3–11.2)	10.2 (8.7–11.3)	0.428
**2-h post load plasma glucose (mmol/L)**	8.7 (8.2–10.5)	8.5 (6.9–10.3)	0.338
**Glycated hemoglobin (HbA1c) (%)**	5.9 (5.6–6.4)	6.1 (5.8–6.5)	0.596
**HbA1c (mmol/mol)**	41 (38–46)	43 (40–48)	0.596
**Fasting plasma insulin (µU/mL)**	13.3 (9.5–21.0)	10.8 (8.7–16.5)	0.541
**1-h post load plasma insulin (µU/mL)**	53.7 (29.2–105.8)	78.7 (54.6–95.6)	0.447
**2-h post load plasma insulin (µU/mL)**	76.9 (44.3–101.9)	75.3 (57.2–115.4)	0.467
**Creatinine (µmol/L)**	61.6 (61.6–70.4)	70.4 (61.6–79.2)	0.289
**Total bilirubin (µmol/L)**	10 (9–15)	12 (7–14)	0.753
**hs-C-reactive protein (nmol/L)**	27.6 (25.7–58.1)	27.6 (22.8–52.4)	0.800
**Aspartate aminotransferase (AST) (U/L)**	29.0 (24.0–39.0)	33 (27–44)	0.427
**Alanine aminotransferase (ALT) (U/L)**	38.5 (36.0–45.0)	42 (33–59)	0.704
**Metformin, *n* (%)**	18 (100)	17 (100)	-
**ACE-I, *n* (%)**	3 (17)	3 (18)	-
**ARBs, *n* (%)**	6 (33)	4 (23)	0.711
**Diuretics, *n* (%)**	5 (28)	3 (18)	0.690
**B-blockers, *n* (%)**	6 (33)	2 (12)	0.228
**CCA, *n* (%)**	0 (0)	0 (0)	-
**Statins, *n* (%)**	2 (11)	3 (18)	0.658
**Fibrates, *n* (%)**	0 (0)	0 (0)	-
**Polyunsaturated fatty acid (PUFA), *n* (%)**	0 (0)	0 (0)	-
**Proton Pump Inhibitors, *n* (%)**	3 (17)	2 (12)	-
**ASA, *n* (%)**	0 (0)	0 (0)	-
**IGF-I (ng/mL)**	85.7 (64.0–111.4)	98.5 (78.7–119.6)	0.322
**Urinary-11-dehydro-thromboxane B_2_ (U-11-dehydro-TXB_2_ ) (pg/mg creatinine)**	1659.5 (1050.0–2589.0)	1833.0 (1170.0–2636.0)	0.947
**Urinary-8-iso-prostaglandin (PG)F_2α_ (U-8-iso-PGF_2α_ )(pg/mg creatinine)**	284.5 (115.0–377.0)	187.0 (129.0–334.0)	0.355
**SAT (cm^2^)**	429.2 (315.7–491.4)	358.9 (262.0–450.6)	0.234
**VAT (cm^2^)**	303.9 (255.3–337.6)	253.0 (162.6–307.5)	0.027
**TNF-α (pg/mL)**	1.01 (0.94–1.44)	1.07 (0.87–1.53)	0.958
**Leptin (pg/mL)**	17.02 (10.8–39.3)	28.02 (13.27–44.73)	0.667
**β-index (pmol^·^min^−2^·m^−2^ Body Surface Area)**	3.41 (2.58–5.08)	4.27 (2.90–5.0)	0.306
**Matsuda-index**	2.9 (2.3–4.4)	2.8 (2.1–4.3)	0.670

Abbreviations: BMI = body mass index, BP = blood pressure, IGT = impaired glucose tolerance, IFG = impaired fasting glucose, CVD = cardiovascular disease, MI = myocardial infarction, TIA = transient ischemic attack, PAD = peripheral artery disease, ACE-I = ACE-inhibitors, ARBs = angiotensin receptor blockers, B-bloc k = beta-blockers, CCA = calcium channel antagonists, ASA = acetylsalicylic acid, IGF-I = insulin-like growth factor I, SAT = subcutaneous-adipose-tissue, VAT = visceral-adipose-tissue, TNF = tumor necrosis factor. Data are median (25th–75th percentile). * Determined by Mann-Whitney or *x*^2^ test, as appropriate.

**Table 2:** Multivariable linear regression analysis (stepwise selection) for Ln-U-11-dehydro-TXB_2._

Predictor Variable	Parameter Estimate	SE	*P*-Value	Partial *R*^2^	Percentage of Variation Relative to 1-SD of Ln-U-11-dehydro-TXB_2_ for 1-SD Change in the Predictor Variable	SD
Ln-U-8-iso-PGF_2α_	0.31	0.10	0.0088	25.3%	+33.2% (95% CI: 9.3% to 57.0%)	0.83
Ln-HbA1c	2.64	0.69	0.0011	16.3%	+51.3% (95% CI: 23.3% to 79.7%)	0.15
Ln-TNF-α	0.58	0.19	0.0075	9.8%	+39.2% (95% CI: 11.7% to 66.7%)	0.53
SAT	0.14	0.06	0.044	16.4%	+25.7% (95% CI: 0.7% to 50.7%)	1.46

SD of Ln-U-11-dehydro-TXB_2_ = 0.78.

**Table 3:** Clinical, biochemical and imaging parameters of obese patients before and after treatment-induced weight loss.

Variable	Pre-Treatment (*n* = 35)	Post-Treatment (*n* = 35)	*p*-Value *
**BMI (kg/m^2^)**	35.5 (32.5–39.2)	33.0 (30.2–36.2)	<0.001
**Waist (cm)**	112.5 (103.0–121.0)	108.0 (99.0–117.0)	<0.001
**Weight (kg)**	99.0 (91.8–111.0)	90.5 (84.3–103)	<0.001
**Systolic BP (mmHg)**	141.0 (127.0–150.0)	133.0 (125.0–145.0)	0.093
**Diastolic BP (mmHg)**	81.0 (77.0–86.0)	80.0 (72.0–85.0)	0.665
**Total cholesterol (mmol/L)**	4.4 (3.8–4.8)	4.1 (3.6–4.5)	0.011
**HDL cholesterol (mmol/L)**	1.1 (1.0–1.4)	1.1 (0.9–1.3)	0.078
**Triglycerides (mmol/L)**	1.3 (0.9–1.5)	1.2 (0.9–1.7)	0.252
**Amylase (U/L)**	64.0 (53.0–75.0)	73.5 (53.0–83.0)	0.024
**Lipase (U/L)**	113.0 (71.0–152.0)	124.0 (100.0–193.0)	0.085
**Fasting plasma glucose (mmol/L)**	5.3 (5.0–5.6)	4.9 (4.6–5.2)	0.0008
**1-h post load plasma glucose (mmol/L)**	10.4 (9.0–11.3)	9.0 (7.7–9.9)	0.0004
**2-h post load plasma glucose (mmol/L)**	8.7 (7.4–10.4)	7.7 (5.1–10.2)	0.0001
**HbA1c (%)**	6.0 (5.7–6.5)	5.6 (5.4–6.0)	<0.0001
**HbA1c (mmol/mol)**	42 (39–48)	38 (36–42)	<0.0001
**Fasting plasma insulin (µU/mL)**	11.5 (8.8–21.0)	9.1 (6.4–11.9)	0.0007
**1-h post load plasma insulin (µU/mL)**	71.8 (37.1–105.8)	65.3 (38.1–113.6)	0.928
**2-h post load plasma insulin (µU/mL)**	75.3 (50.4–107.7)	55.9 (31.4–113.1)	0.171
**Creatinine (µmol/L)**	67.8 (59.0–79.2)	68.6 (60.7–79.2)	0.862
**Total bilirubin (µmol/L)**	11 (7–15)	10 (8–13)	0.489
**hs-C-reactive protein (nmol/L)**	27.6 (23.8–58.1) 48.3 ± 7.6 *	27.6 (12.4–43.8) 33.8 ± 5.3 *	0.0006
**AST (U/L)**	32.0 (24.0–39.0)	23.0 (20.0–28.0)	<0.0001
**ALT (U/L)**	40.0 (34.0–58.0)	33.0 (27.0–41.0)	<0.0001
**IGF-I (ng/mL)**	90.2 (68.6–111.9)	100.0 (75.8–128.7)	0.035
**U-11-dehydro-TXB_2_ (pg/mg creatinine)**	1710 (1050–2636)	1080 (619–1569)	<0.0001
**U-8-iso-PGF_2α_ (pg/mg creatinine)**	264 (115–377)	165 (95–324)	0.035
**SAT (cm^2^)**	390.9 (297.7–485.1)	329.1 (206.4–435.5)	<0.0001
**VAT (cm^2^)**	262.1 (180.5–336.5)	238.0 (174.7–292.9)	<0.0001
**TNF-α (pg/mL)**	1.06 (0.88–1.53)	1.14 (0.84–1.36)	0.936
**Leptin (pg/mL)**	18.7 (10.8–44.7)	12.5 (7.7–28.7)	<0.0001
**β-index (pmol^.^min^−^2·m^−2^ BSA)**	3.74 (2.62–5.08)	4.78 (3.40–5.22)	0.0033
**Matsuda-index**	2.9 (2.1–4.4)	4.0 (3.1–5.2)	0.0210

Abbreviations: BMI = body mass index, BP = blood pressure, HDL = high density lipoprotein, hs = high sensitivity, AST = aspartate aminotransferase, ALT = alanine aminotransferase, U-11-dehydro-TXB_2_ = urinary-11-dehydro-tromboxane B_2_, IGF-I = insulin-like growth factor I, SAT = subcutaneous-adipose-tissue, VAT = visceral-adipose-tissue, TNF = tumor necrosis factor. Data are median (25th–75th percentile). Determined by paired t-test. * Mean ± SD.

**Table 4:** Correlations between change in platelet activation and change in glycometabolic variables.

	Delta VAT	Delta SAT	Delta -index	Delta Matsuda Index	Delta leptin	Delta FPG	Delta 1-h-PPG	Delta 2-h-PPG	Delta hs-CRP	Delta HbA1c	Delta IGF-I	Delta TNF-α	Delta U-8-iso-PGF_2α_
**Delta U-11-dehydro-TXB_2_**	*r* = −0.199	*r* = −0.110	*r* = 0.150	*r* = 0.124	*r* = 0.380	*r* = −0.07	*r* = 0.010	*r* = 0.097	*r* = 0.099	*r* = −0.136	*r* = 0.069	*r* = 0.561	*r* = 0.357
	*p* = 0.250	*p* = 0.527	*p* = 0.388	*p* = 0.476	*p* = 0.024	*p* = 0.680	*p* = 0.952	*p* = 0.576	*p* = 0.601	*p* = 0.432	*p* = 0.691	*p* = 0.001	*p* = 0.034

Abbreviations: FPG = fasting plasma glucose, 1h-PPG= 1-h post-load plasma glucose, U-11-dehydro-TXB_2_ = urinary-11-dehydro-tromboxane B_2_, IGF-I = insulin-like growth factor I, SAT = subcutaneous-adipose-tissue, VAT = visceral-adipose-tissue, TNF = tumor necrosis factor.

**Table 5:** Multivariable linear regression analysis for delta Ln- U-11-dehydro-TXB_2._

Predictor Variable	Standardized Parameter Estimate	SE	*P*-value	Partial *R*^2^	Percentage of Variation Relative to 1-SD of Ln-U-11-Dehydro-TXB_2_ for 1-SD Change in the Predictor Variable	SD
Delta Ln-U-8-iso-PGF_2α_	0.78	0.23	0.0042	23.1%	+88.1% (95%CI: 5.5% to 92.6%)	0.62
Delta TNF-α	0.10	0.04	0.0294	6.8%	+49.1% (95%CI: 31.8% to 144.3%)	2.63

SD of delta Ln-U-11-dehydro-TXB_2_ = 0.55.
